# Highly sensitive fluorometric method based on nitrogen-doped carbon dot clusters for tartrazine determination in cookies samples

**DOI:** 10.3906/kim-1903-28

**Published:** 2020-02-11

**Authors:** Abidin GÜMRÜKÇÜOĞLU, Aysel BAŞOĞLU, Sevgi BAŞOĞLU, Saliha BAŞOĞLU, Meryem BAŞOĞLU, Miraç BAŞOĞLU, Ümmühan BAŞOĞLU

**Affiliations:** 1 Department of Chemistry, Faculty of Arts and Sciences, Karadeniz Technical University, Trabzon Turkey; 2 Department of Occupational Health and Safety, Faculty of Health Sciences, Gümüşhane University, Gümüşhane Turkey; 3 Organic Farming Management, Çumra School of Applied Sciences, Selçuk University, Konya Turkey; 4 Advanced Technology Research & Application Center, Çumra Vocational School, Selçuk University, Konya Turkey

**Keywords:** Carbon dot, amorphous, fluorescence, tartrazine, determination method, cookie

## Abstract

Nitrogen-doped carbon nanodots (CDs) were prepared via the solvothermal method, using urea and triethylene glycol as the starting materials. The as-prepared CDs had individual diameters of approximately 100 nm and were in clusters of different sizes. The surface composition and optical properties of the as-prepared CDs were characterized. They exhibited multicolor emission properties in the visible range when excited with a wide wavelength range. The aqueous solution of the CDs was used in highly sensitive tartrazine determination. The fluorescence quenching of the CDs was in a linear relationship with the concentrations of tartrazine in the range of 0.5–30.0 μM. The detection limit of the assay was 0.18 μM. Acceptable recovery results were obtained via spike-recovery experiments on cookie samples.

## 1. Introduction

Orange-colored tartrazine is a commonly used synthetic food colorant in drugs, food, cosmetics, and the pharmaceutical industry. Azo-dye tartrazine is known as FD&C Yellow No. 5, C.I. No. 19140, Food Yellow No. 4, and E102 [1]. Its excess utilization in food may cause adverse health effects including allergic reactions, migraines, eczema, anxiety, oxidative stress, and DNA damage [2,3]. Due to their impact on health, the use of food dyes in foodstuffs is also monitored legally [4]. Therefore, efficient, rapid, simple, and suitable analytical techniques are needed for the assurance of health and food safety. To date, various determination techniques have been applied for tartrazine dye, such as chromatography, mass spectrometry, capillary electrophoresis, and electrochemical methods [1,2]. However, these methods require sophisticated equipment and time-consuming sample preparation steps that may not be suitable for routine detection. Therefore, there is a significant need to develop economic, simple, and ecofriendly detection strategies for food colorants, including tartrazine dye.

Nanomaterials have opened new frontiers with a wide range of applications, such as the utilization of biosensors for environmental and food safety monitoring, disease detection, drug discovery, and point-ofcare monitoring [5]. Carbon nanodots (CDs) are usually nanoparticles that have a size below 10 nm [6]. However, CDs larger than 10 nm have also been reported in the literature [7]. These nanostructures have attracted attention due to their useful optical properties, such as high quantum yield and long wavelength emissions [5,8,9]. Since discovered, CDs have intensively been used in many application areas in the fields of medical diagnosis, catalysis, bioimaging, and sensors [10,11]. Easy and green synthesis, low toxicity, excellent biocompatibility, good photostability, good water solubility, and low cost are some of the unique benefits of CDs [12]. The emission characteristics of CDs with distinct optical properties change according to their size due to quantum confinement effects [8]. Any simple compounds, such as citric acid, urea, and ascorbic acid, can be used as starting materials to prepare CDs [13]. Furthermore, materials including foods, plants, candle soot, and waste have also been used as carbon sources for the synthesis of CDs [14]. The extraction of CDs from some food sources and food wastes are also applicable [15].

Synthesis methods for carbon dots are basically classified into top-down and bottom-up methods. In the top-down method, CDs are prepared from a large carbon structure such as a carbon nanotube and graphite using laser ablation, arc-discharge, and electrochemical methods, while in the bottom-up approach, CDs are synthesized from molecular precursors, such as citric acid and urea, using hydrothermal, microwave-assisted methods, combustion, or thermal routes [8]. The optical properties of CDs may be improved via heteroatom doping or surface passivation techniques [16]. With its atomic size comparable to that of carbon atoms, the nitrogen atom is considered as an important element for the doping of carbon dots [17]. Wang et al. synthesized high photoluminescence quantum yield nitrogen (N)-doped carbon dots using precursors of urea and diethylene glycol by microwave pyrolysis to determine iron(III) [18].

Recently, fluorescent carbon dots obtained from aloe were developed for the selective detection of tartrazine in food samples of candy, steamed buns, and honey by a few researchers [19]. In a similar way, luminescent CDs from citrus peels were used for tartrazine sensing in different food matrices, such as ice cream, juice, and energy drinks [20]. There was no application in cookie matrices among these studies. Cookie matrix is different from the matrices in these previous studies. Despite the existence of these few studies on the utilization of CDs in the determination of synthetic food dyes, to the best of our knowledge, there have been no studies on the utilization of doped CDs for the determination of tartrazine in cookies. Herein, we developed a fluorescence probe for the determination of tartrazine in cookie matrix, for the first time, using N-doped CDs synthesized from molecular precursors of urea and triethylene glycol (TEG) via the solvothermal method using a domestic microwave oven.

## 2. Materials and methods

### 2.1. Instrumentation

All fluorescent measurements were performed on a PTI QM-4 spectrofluorometer with a slit width of 1.0 nm in a 1-cm quartz cell. UV-Vis absorption spectra of CDs were recorded on an Analytik Jena Specord 210 spectrophotometer (Analytik Jena AG, Jena, Germany). Transmission electron microscopy (TEM) studies for the morphological characterization of CDs were carried out using an FEI TALOS F200S TEM 200 kV (Thermo Fisher Scientific, Waltham, MA, USA). Next, 1 mL of the aqueous CD solution was diluted with ethanol and ultrasonicated before being placed on a carbon-coated copper grid and dried at room temperature. The FTIR spectra were recorded on a PerkinElmer 1600 spectrophotometer (PerkinElmer, Inc., Waltham, MA, USA) in the range of 550–4000 cm^-1^ after the CDs had been freeze-dried. X-ray photoelectron spectroscopy (XPS) was conducted using an ESCALAB MK II X-ray photoelectron spectrometer (Thermo Fisher Scientific). For the analysis, several drops of the CD solution were applied to a thoroughly cleaned silicon wafer and dried in a vacuum oven. X-ray diffraction (XRD) patterns were obtained with a Bruker D2-phaser diffractometer using CuKα radiation (λ = 1.5418 Å). For the XRD analysis, the sample was prepared by dropping the CD solution onto a cleaned glass wafer. Deionized water in the analytical measurements was obtained using the Sartorius Milli-Q system (arium 611UV; Sartorius AG, Göttingen, Germany).

### 2.2. Reagents

Urea and TEG, purchased from Merck (Darmstadt, Germany), were used to prepare the CDs. Quinine sulfate (Sigma-Aldrich, St. Louis, MO, USA) was used as the fluorescence standard compound. Sunset yellow, allura red, quinoline yellow, and tartrazine were all purchased from Sigma-Aldrich. The stock solutions of tartrazine and allura red were prepared in water. Ethanol was used to prepare the stock solutions of sunset yellow and quinoline yellow. The working solutions were prepared by an appropriate dilution of the stock solution (1000 μM).

### 2.3. Samples

Cookie samples were purchased from local markets in Trabzon, Turkey. Extraction of tartrazine in the samples was carried out with water. Next, 2.0 g of the spiked cookie sample and the original cookie sample, in 100 mL of water, was held in an ultrasonic bath for 15 min. After the treatment, the mixture was shaken at 140 rpm for 30 min, and then filtered through a 0.20-μm membrane. The filtrate of the spiked sample was used as the sample solution. The filtrate of the original sample was used as the matrix solution.

### 2.4. Preparation of the carbon dots

Fluorescent CDs were synthesized using the solvothermal method. Therefore, 1 g of urea was added to 10 mL of TEG, and then ultrasonicated for 5 min to form a transparent solution. The solution was kept in a domestic microwave oven (700 W, Sinbo, Turkey) for 10 min. The color of the solution changing from colorless to yellow was an indicator of the formation of CDs. The yellow solution was used by 1/1250 (V/V) dilution with deionized water in the fluorescence measurements.

### 2.5. Determination of the quantum yield

To determine the relative fluorescence quantum yield of the CDs (Φ_x_), quinine sulfate dissolved in 0.1 M H2 SO4 was used as the reference (quantum yield is 0.546) [21]. The emission (excited at 320 nm) and absorption spectra of the CDs and quinine sulfate at different concentrations were recorded. The absorbance values at 320 nm were then plotted on the X-axis and the areas of the emission spectra were plotted on the Y-axis to determine the slope of the curve. The quantum yield was then determined using Eq. (1):

(1)Φx=ΦR(mx/mR)(ηx2/ηR2)

Here, Φ _x_ and Φ _R_ represent the quantum yield of the CDs and quinine sulfate, respectively. m _x_ and m _R_ represent the slope of the curve related to the CDs and the reference, respectively. η is the refractive index of the solvent. The refractive index of quinine sulfate (1.33) equals those of the CDs dissolved in deionized water. The experimental details are given in the Supplemental information.

### 2.6. Tartrazine determination

The method was based on fluorescence quenching of the CDs with tartrazine. A kind of standard addition method was used to determine the tartrazine amount in food samples. A similar standard addition method was previously used in the fluorometric determination of a banned synthetic food dye, Sudan I [22]. The experimental details are given in the Supplemental information.

## 3. Results and discussion

### 3.1. Synthesis and characterization of the CDs

**Figure 1:**
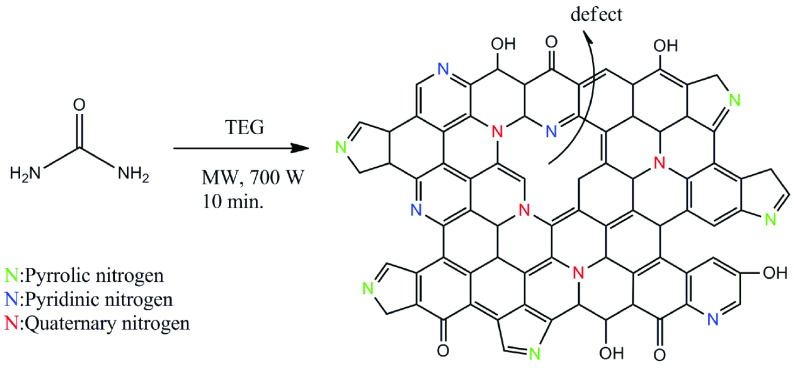
Synthesis and structural representation of the nitrogen-doped CDs in this study.

A 1-step synthesis procedure involving the solvothermal method was performed to prepare the CDs using a domestic microwave oven (Figure 1).

**Figure 2:**
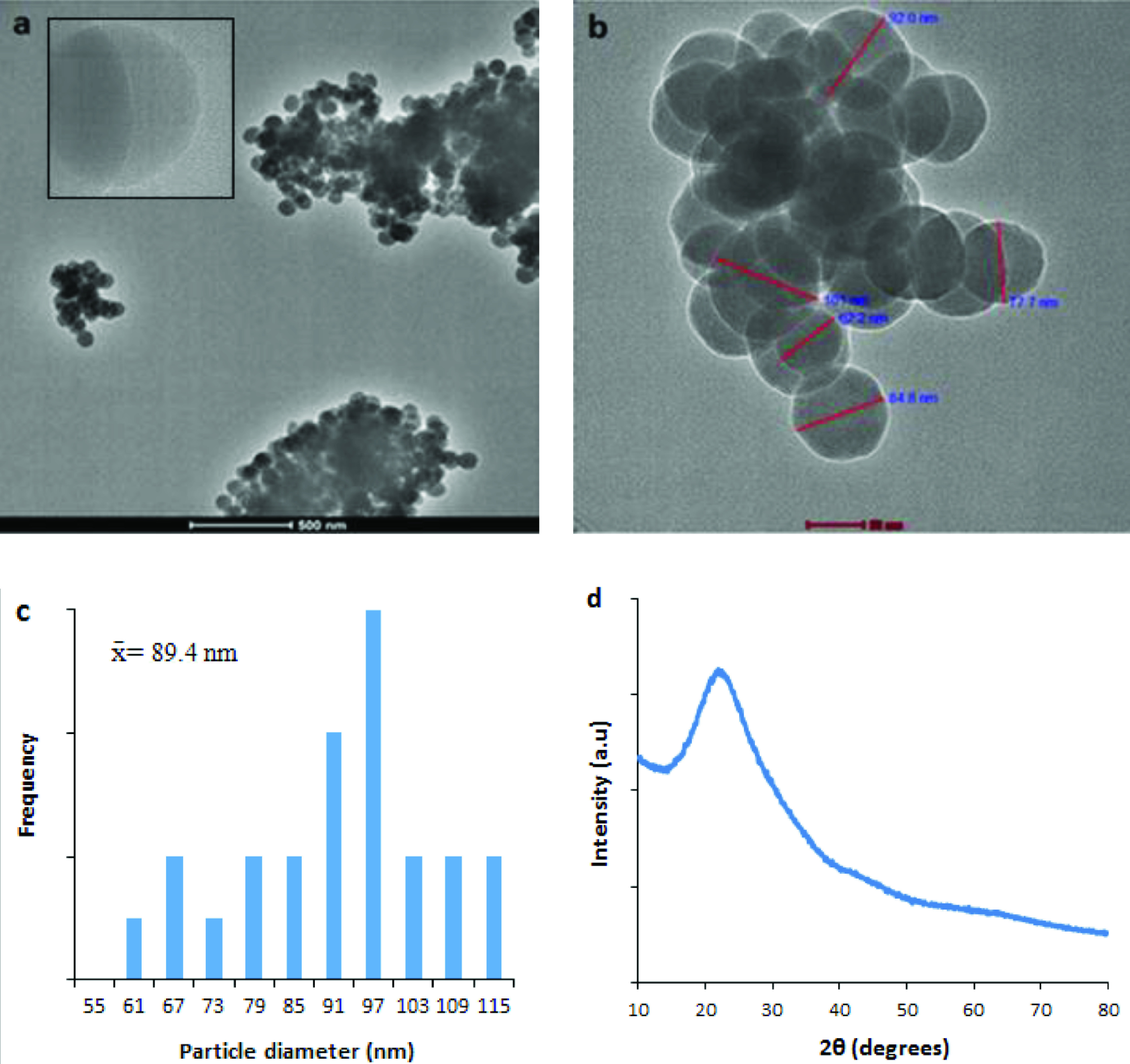
TEM images, particle distributions, and XRD profile of the CDs. a) CD nanoclusters in micro size, inset: high magnification image showing 2 overlapped CDs without the signature of any fringe; b) CD nanocluster with different particle sizes; c) particle size distribution; d) XRD profile of the CDs.

The CDs were obtained as a viscous liquid. Their morphology and microstructure were investigated via high-resolution transmission electron microscopy. The TEM image of the CDs in the microscale showed clusters in different shapes, just like carbon black (Figure 2a). A few CDs with similar aggregation have been reported in the literature [23–26]. When the image of a cluster was magnified, it was seen that CDs with different diameters had almost spherical structures (Figure 2b). The average diameter of the particles in Figure 2b was determined as 89.4 ± 14.2 nm. There was no signature of fringes in the high-magnification image of the CDs, which showed the amorphous structures of the CDs (Figure 2a, inset). Diameters of the amorphous CDs varied between ∼61 and ∼113 nm, as shown in the histogram (Figure 2c). The broad hump centered at ∼2θ = 22◦ in the XRD profile shown in Figure 2d reflects that the material had a defective structural order, namely weak graphitic crystallinity. This kind of hump has been widely reported for XRD patterns of amorphous carbon [27–30]. The interlayer spacing of ∼0.4 nm calculated from Bragg’s law was larger than that of the graphite with an interlayer spacing of 0.3 nm. This result can be explained by the presence of functional groups, such as O-H and C=O, on the surface of the weakly graphitic sheets within the CDs [30,31]. After purification of the CDs with dialysis, the TEM images were obtained (Figure S1, left). There were no CD clusters in the images and good dispersion of the CDs was observed. The amorphous structure of the CDs is supported by the selected-area electron diffraction (SAED) image of an individual CD as shown in Figure S1 (right).

**Figure 3:**
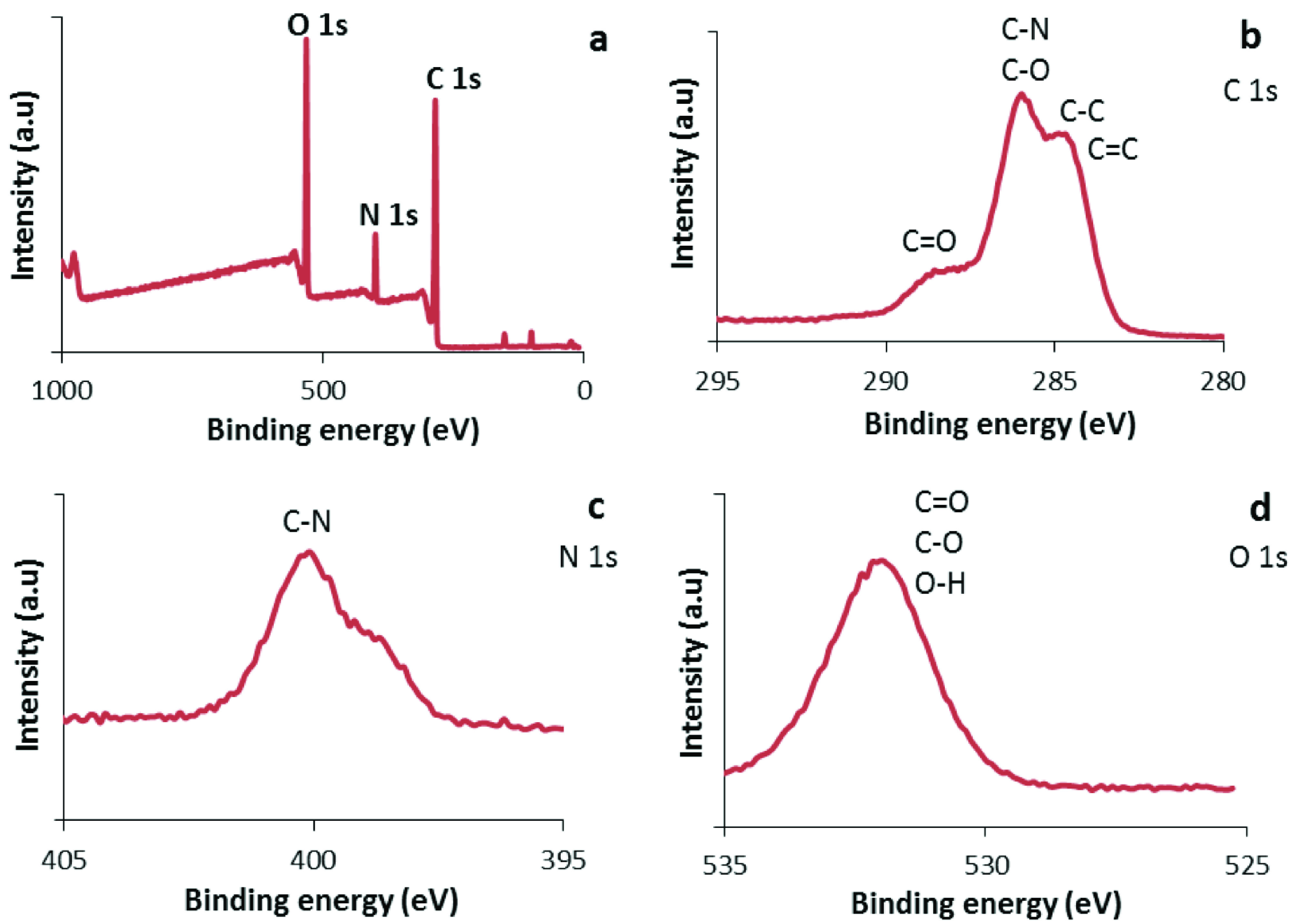
XPS full spectrum for the CDs. a) C 1s, b) N 1s, c) O 1s, d): spectra.

The XPS and FTIR spectra provided detailed information about the functional groups on the surface of the CDs. XPS was used to characterize the elemental surface composition of the CDs. Figure 3a shows XPS spectra with 3 peaks centered at 285.1 eV (C 1s), 400.1 eV (N 1s), and 532.4 eV (O 1s), indicating the presence of carbon, nitrogen, and oxygen in the structure. These 3 main peaks were examined in detail. Three peak groups at 284.5–285.4, 286.0, and 288.4–289.1 eV in the C 1s spectra were assigned to the C=C/C–C, C–O/C–N, and C=O groups, respectively (Figure 3b) [18,19]. The N 1s band consisted of 3 types of peaks: pyridinic, pyrrolic, and quaternary nitrogen, with bond energies between 397.8 and 401.9 eV (Figure 3c) [32,33]. The main peak was at 400.1 eV, which was attributed to a pyrrolic C-N bond [33]. These peaks indicated that nitrogen was doped on the surface of the CDs [33]. Namely, the dopant nitrogen atoms were inserted in the defect area or the surface of the slightly graphitic structure. Figure 3d shows the O 1s spectra with a broad peak between 534.2 and 530.3 eV, which can be attributed to C=O/O-H and C-O [27]. Specifically, the peak centered at 532.3 eV resulted from the OH bond [23].

**Figure 4:**
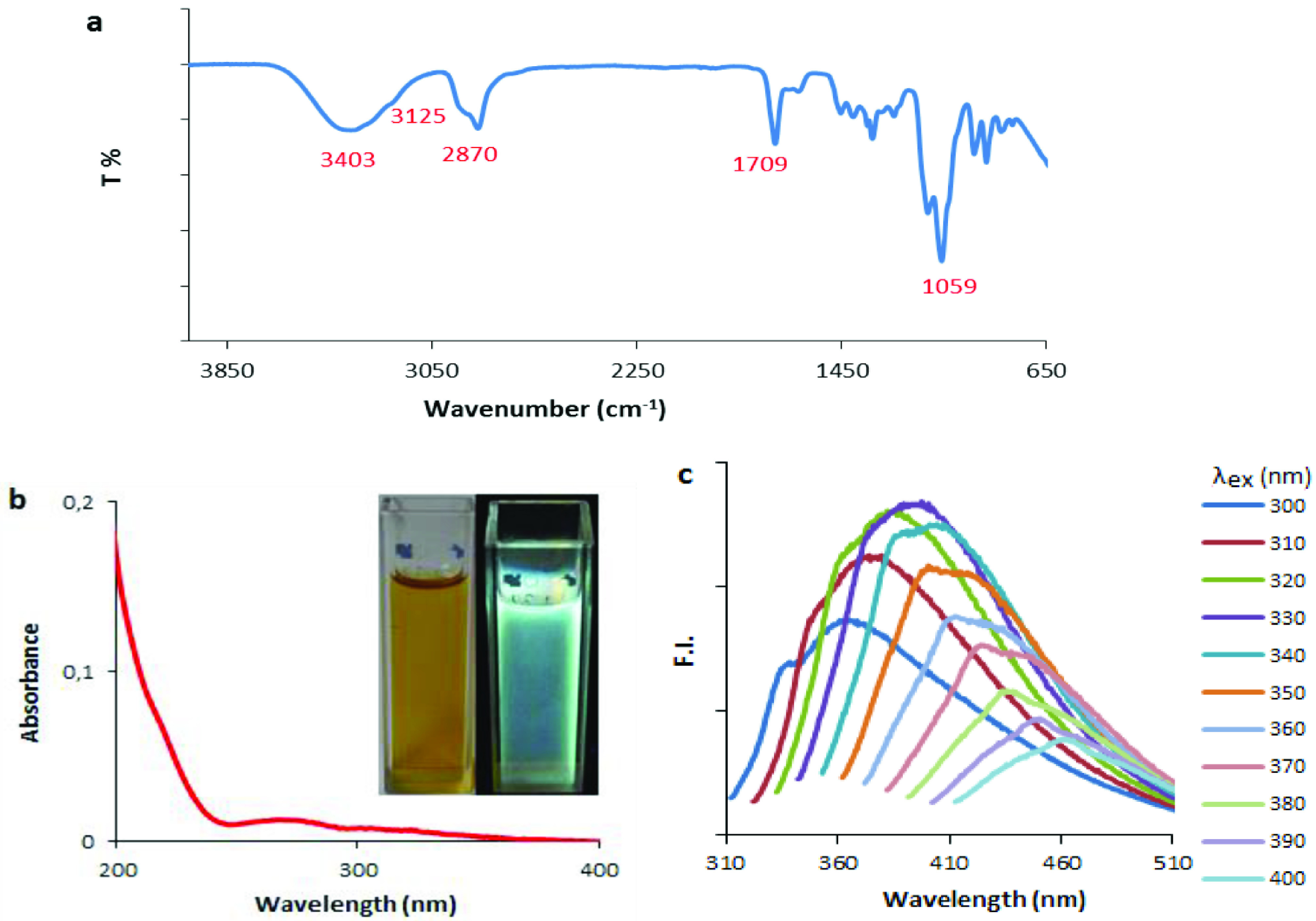
Optical properties of the CDs. a) FTIR spectra, b) UV-Vis absorption spectra (inset: under ambient and UV light), c) emission spectra at different excitation wavelengths.

To further detect the functional groups of the CDs, FTIR spectroscopy was used, as shown in Figure 4a. The peak at 3403 cmcm^-1^ was attributed to the O-H stretching vibration. The band at 2870 cmcm^-1^ was attributed to aliphatic C-H bonds. Aromatic C-H stretching vibration was observed 3125 cmcm^-1^ in the FTIR spectra, suggesting that a graphitic structure was contained in the CDs. This result was compatible with the XPS analysis. In the XPS spectra, C=C binding energy was observed at about 284.5 eV [23]. In the FTIR spectra, the absorption peak at 1709 cmcm^-1^ was assigned to the stretching vibration of C=O coming from urea under solvothermal treatment in TEG. The sharp peak at around 1059 cmcm^-1^ contributed to the stretching vibration bands of C-O and C-N. These vibration bands suggested that the surface of the CDs was passivated by surface groups during the carbonization process of urea. Based on all of the characterization data, the proposed structure for the CDs is given in Figure 1.

The photoluminescence (PL) of CDs is one of the most important features that enables their use for analytical purposes [13,16]. Both crystalline and amorphous CDs exhibit PL properties [30]. CDs synthesized by the bottom-up method would generally have an amorphous structure [30]. Although the mechanism of PL of both CD species is not well understood, it is thought that the surface defects and emissive energy traps are primarily responsible for the fluorescence characteristics [34]. The band at 265 nm in the absorption spectra of CDs is related to π–π* transitions of the C=O bond. The aspect of the spectra in Figure 4b was very characteristic for CDs [35]. The aqueous solution of the as-prepared CDs was clear yellow in color under ambient light, while it showed strong blue fluorescence when excited at 365 nm (Figure 4b, inset). Specifically, the multicolor properties of amorphous CDs could be explained by abundant surface defects and high entropy states in amorphous nanostructures [27]. Figure 4c shows the excitation wavelength-dependent emission properties of the CDs. As can be seen from Figure 4, different emission maxima were obtained in the blue region with increasing excitation wavelength. This type of excitation wavelength-dependent emission has been generally observed with CDs in the literature [5,6].

The fluorescence quantum yield of the CDs was calculated according to a known method [36]. The quantum yield was determined as 11% at an excitation wavelength of 320 nm using quinine as a reference (Figure S2). Absorbance in the 1-cm fluorescence cuvette should never exceed 0.01 at the excitation wavelength to minimize the effects of reabsorption. The high quantum yield results from nitrogen doping into the nanostructure [32,37].

### 3.2. Interaction with food dyes

The interaction of tartrazine, sunset yellow, allura red, and quinoline yellow with the as-prepared CDs based on urea was investigated via the fluorometric measurements.

**Figure 5:**
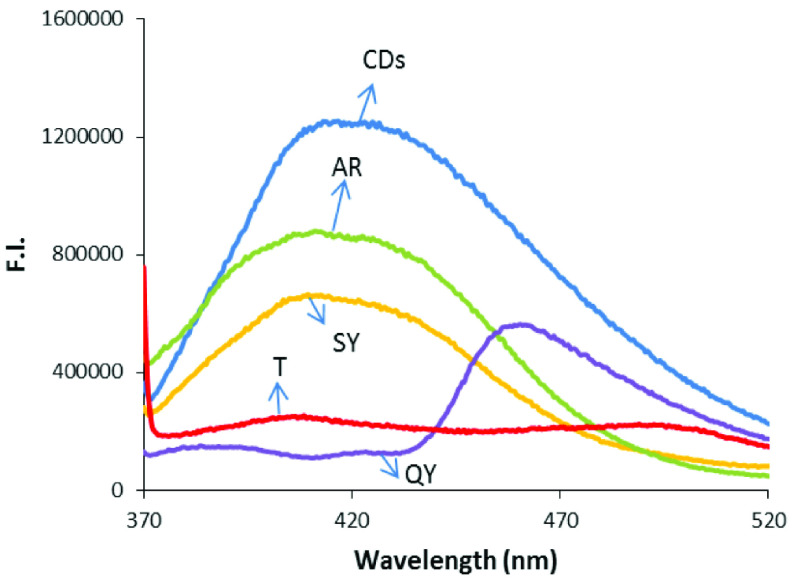
Effect of the food dyes on the fluorescence spectra of the CDs. Excitation wavelength: 360 nm. AR: alurra red, SY: sunset yellow, T: tartrazine, QY: quinoline yellow.

Figure 5 shows the changes in the fluorescence spectra of the CDs with the food dyes, all of which caused fluorescence quenching in the spectra. However, the prepared calibration graphs did not work to determine these food dyes in real samples, except for tartrazine. Consequently, it was determined that the CDs were suitable for the determination of only tartrazine among the tested food dyes. The quenching mechanism of the CDs with tartrazine was investigated using Stern–Volmer analysis (Figure S5). The results are given in the Supplemental information.

### 3.3. Tartrazine determination

**Figure 6:**
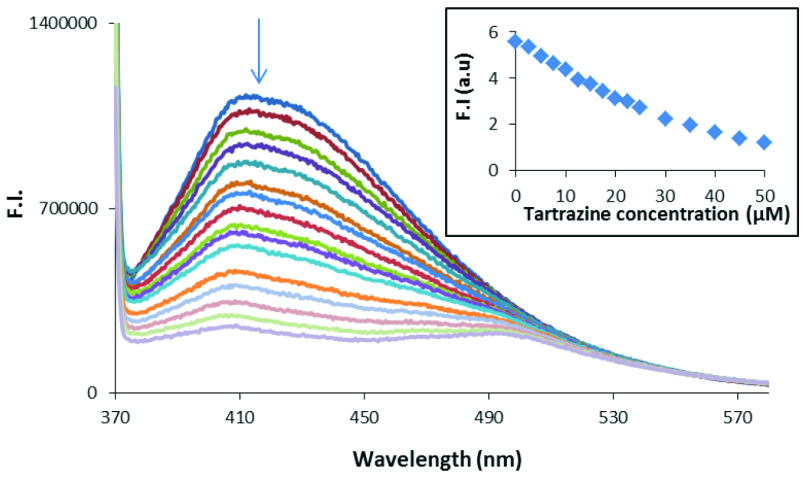
Fluorescence quenching in the spectra of the CDs with increasing tartrazine concentrations. Inset: Calibration graph for tartrazine determination.

Figure 6 shows the change in the fluorescence spectra of the CDs with increasing tartrazine concentration.

As seen in Figure 6, there was regular fluorescence quenching in all of the spectra while the tartrazine concentration increased. From the fluorescence intensity at 425 nm, an external calibration graph was prepared to determine tartrazine (Figure 6, inset). As seen in Figure 6 (inset), there was a deviation from Beer’s law after 30 μM of tartrazine. The linear range was between 0.5 μM and 30 μM. The external calibration line was tested to determine tartrazine in the cookie samples. However, satisfactory accuracy values could not be achieved using the external calibration graph. Therefore, a kind of standard addition method was used in the tartrazine determination. The experimental details are given in the Supplemental information (Figures S3 and S4). This kind of calibration procedure in fluorometric methods was previously used [22].

The analytical performance data for the developed method are presented in Table 1.

**Table 1:** Analytical performance data of the proposed method for tartrazine determination.

Excitation wavelength (nm)	360
Emission wavelength (nm)	425
LOD (μM)	0.18
LOQ (μM)	0.54
Linear range (μM)	0.5–30.0
Solvent	Water
Time before measurement	1–2 min
The correlation coefficient (R ^2^ )	0.9934
Intraday precision (RSD%, N = 3, for 2.5 mg/L)	2.4
Interday precision (RSD%, N = 3, for 2.5 mg/L)	1.4

RSD%: Relative standard deviation, LOD: limit of detection, LOQ: limit of quantification.

As seen from Table 1, the correlation coefficient was 0.9934, indicating good linearity. To calculate the limit of detection (LOD), 3 times the standard deviation was divided by the slope of the calibration line. To determine the standard deviation in these experiments, 11 measurements of the blank response were carried out. The limit of quantification (LOQ) was determined as 3 times the LOD.

The accuracy of the method was verified by the analysis of the spiked cookie samples at different concentration levels. The standard addition method was applied to 4 different concentration levels in the linear range, and recovery was between 99.8% and 96.9% for these concentration levels (Table 2). The results showed that the proposed method can be applied for the determination of tartrazine in cookies samples.

**Table 2:** Recovery studies of tartrazine in the cookie samples.

Added (mg/L)	Found (mg/L)	Amount in the sample (mg/L) ± RSD%	R%
2.5	5.0	2.5 ± 4.0	96.9
5	7.5	2.5 ± 0.6	99.4
6	8.5	2.5 ± 0.2	95.0

R%: Recovery%.

The cookie samples were also analyzed using the standard high-performance liquid chromatography (HPLC) method [38]. The results are compared in Table 3. Student’s t-test was used to statistically analyze the results. The calculated Student’s t-value (0.68) was less than the theoretical value (4.30) at a confidence level of 95%. Therefore, the statistical calculation showed no significant difference between the results of the proposed method and the standard method [38].

**Table 3:** Comparison of the tartrazine results in the cookie samples (N = 3).

Proposed method (mg/kg)	RSD%	R%	Standard method (mg/kg) [38]	MU ^a^	R%
255.7	4.0	97.1	262.0	13.1	98.8

^a^ Measurement uncertainty.

### 3.4. Comparison with other methods

A comparison of the proposed method and some other methods for the determination of tartrazine is given in Table 4.

**Table 4:** Comparison of some tartrazine determination methods in the literature.

Method	Reagent	Sample	LR (μM)	LOD (μM)	Ref.
FL	CDs (citrus peels)	Ice cream, juice, energy drink	0.6–23.5	0.2	[20]
FL	CDs (aloe)	Candy, honey, steamed buns	0.25–32.5	0.07	[19]
HPLC	PA	Juice, saffron, rice, cookie	0.93–4.68	0.07	[43]
FL	CDs (urea)	Cookie	0.5–30.0	0.18	TS

FL: Fluorescence, LR: linear range, TS: this study, PA: polyamide adsorbent.

Tartrazine in food is usually determined using spectrophotometric, electrochemical, and chromatographic methods [39–42]. However, there are few chromatographic methods used for the determination of tartrazine in cookies [43]. As seen in Table 3, the linear range of the method proposed in the literature was quite narrow (0.93–4.68 μM) [43]. Moreover, the recovery was low (59%) [43]. There are 2 studies regarding the determination of tartrazine by CDs. Citrus peels and aloe, as a carbon source, were used to prepare CDs in these studies, respectively [19,20]. Xu et al. used CDs from aloe to determine tartrazine in steamed buns, as well as honey and candy samples [19]. However, they used a Teflon-lined autoclave and it was necessary to heat the carbon source at 180 ◦ C for 11 h to obtain the CDs. Similarly, in the other study, citrus peels were heated in a furnace at 180 ◦ C for 2 h to prepare the CDs [20]. Moreover, no application was made in a matrix similar to a cookie sample in these studies [19,20]. The present study was the first to use CDs for tartrazine determination in a cookie matrix. The preparation of the CDs was simple and fast (10 min) and needed only a domestic microwave oven. Therefore, this simple and fast method will fill the gap in the literature for the determination of tartrazine in cookie matrices. Moreover, the proposed method to determine tartrazine was inexpensive and fast when compared to other expensive and time-consuming HPLC methods in the literature.

Supplementary Materials
