# Supplementary material for: Highly sensitive fluorometric method based on nitrogen-doped carbon dot clusters for tartrazine determination in cookies samples
Source: Turk J Chem. 2020 Feb 11;44(1):99–111. doi: 10.3906/kim-1903-28 (PMC7751817; doi:10.3906/kim-1903-28)
Supplement: Supplementary file 1 — Supplementary Materials [file turkjchem-44-99-sup001.pdf]

Supplementary information

1. Characterization of the CDs

The CDs were purified with a 6000-Da molecular weight cut-off dialysis bag. After 24 h of dialysis against ultrapure water, the solution outside of the dialysis bag was collected and the ultrasonic process was applied to the aqueous solution for 20 min. The TEM images of the final solution were obtained (Figure S1, left). As seen in Figure S1 (left), a good particle size distribution was observed below ∼10 nm. Moreover, the SAED images of the individual dots were obtained to see the crystallinity of the CDs (Figure S1, right). As seen in Figure S1 (right), the diffraction rings were observed in the SAED image, resulting from the amorphous structure of the CDs.

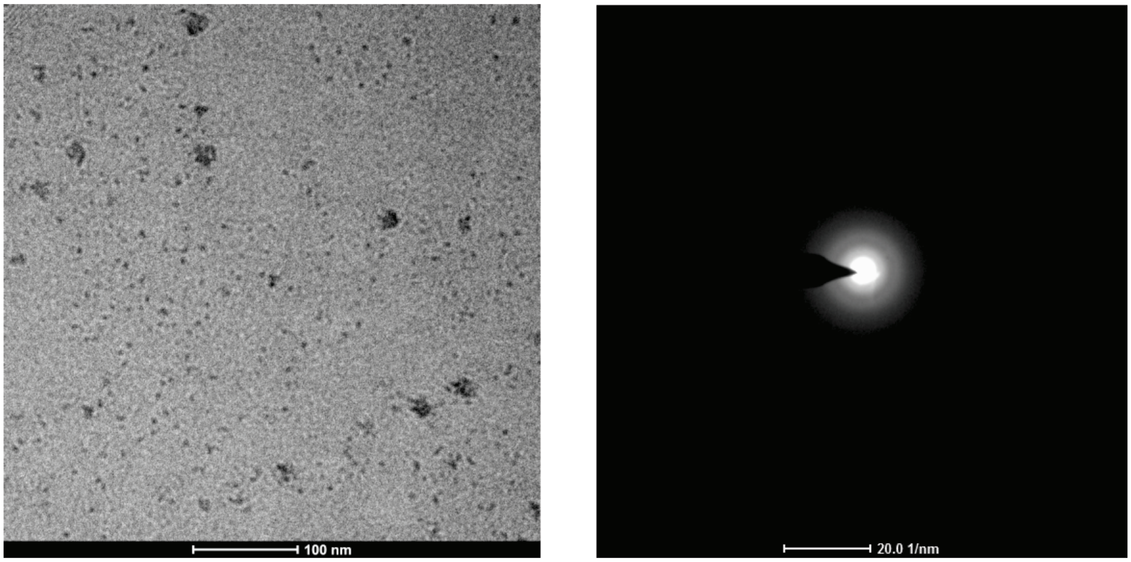

Figure S1. TEM image of the CDs after the dialysis and ultrasonication process (left) and the SAED image of an individual CD (right).

2. The proposed method

CDs (2 mL) and an aliquot of the matrix solution (100 μL) were added to all of the tubes. A certain volume of the sample solution was added to all of the tubes, except for the first tube, to complete the final volume to 4 mL. The difference between the fluorescence intensities of the first and the second tubes was related to the tartrazine concentration in the sample solution. Fluorescence intensity of all solutions was measured at 425 nm. Using Eq. (1), the tartrazine concentration was calculated.

$$C_x = (F_0 - F_1)/m \tag{1}$$

In Eq. (1), C_x is the tartrazine concentration of the spiked sample in the tubes. Fluorescence intensities of the first and second tubes are represented by F_0 and F_1, respectively. m is the slope of the standard addition graph. Figures S3 and S4 show the fluorescence quenching of the solution in the tubes with increasing tartrazine amount and the standard addition graph for the determination of tartrazine (2.8 μM) in the spiked cookie sample, respectively.

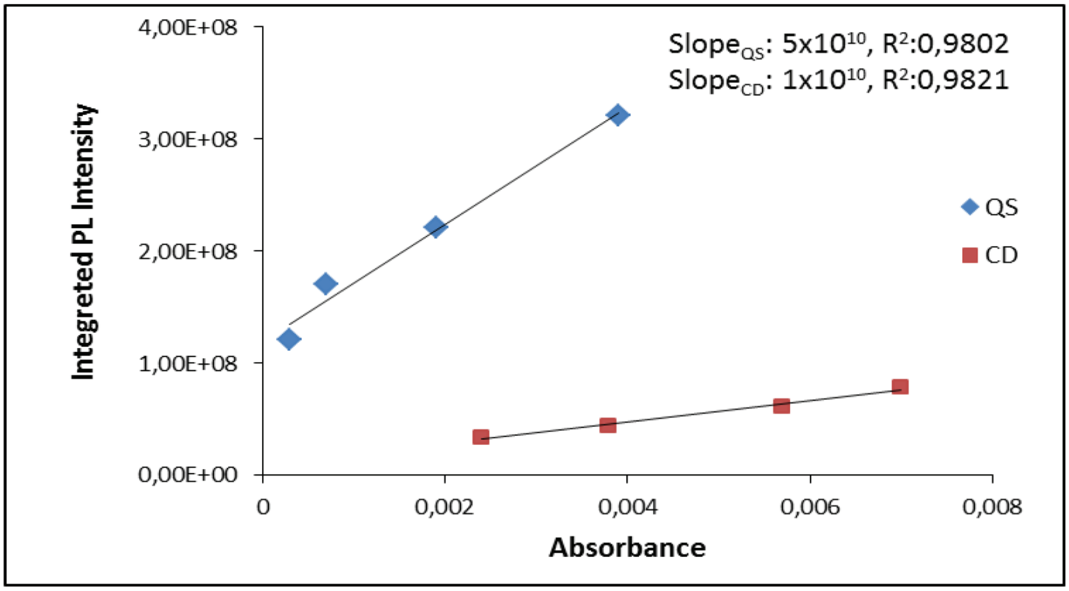

Figure S2. Integrated PL intensity and absorbance of the CDs and QS to determine the quantum yield (excited at 320 nm).

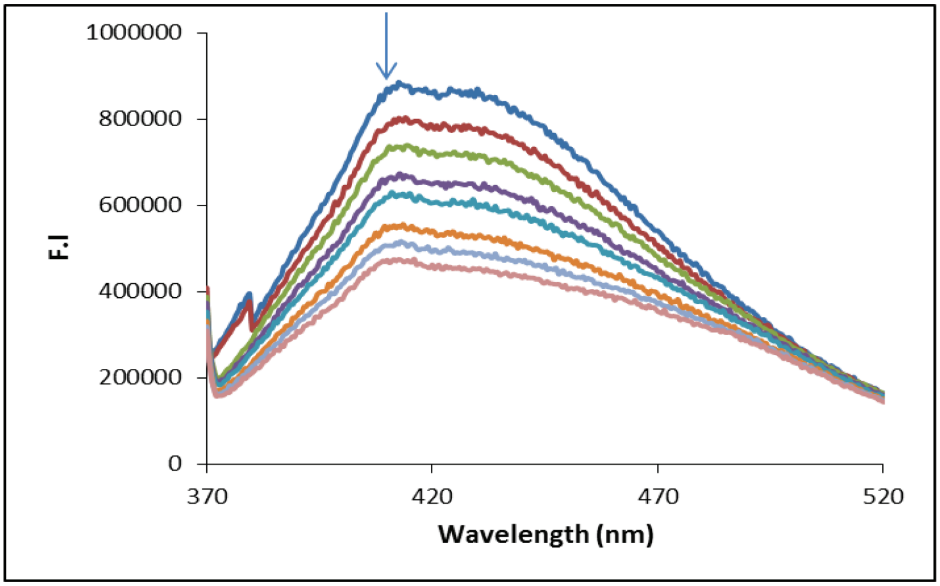

Figure S3. Quenching in the fluorescence spectra of the CDs with increasing tartrazine concentrations.

3. Quenching mechanism

The Stern–Volmer relationship can explain the quenching mechanism of the fluorescent CDs by tartrazine as a quencher, as seen in Eq. (2).

$$I_0/I = 1 + K_{sv}[Q] \tag{2}$$

In Eq. (2), K_{sv} is the Stern–Volmer quenching constant and I_0 and I are the fluorescence intensities in the absence and presence of the quencher, respectively. [Q] represents the concentration of the quencher. When the quenching mechanism fits the Stern–Volmer equation, the plot of I_0/I versus the quencher molar concentration gives a straight line. In this case, the slope is K_{sv} and the Y-axis intercept should be 1. Therefore,

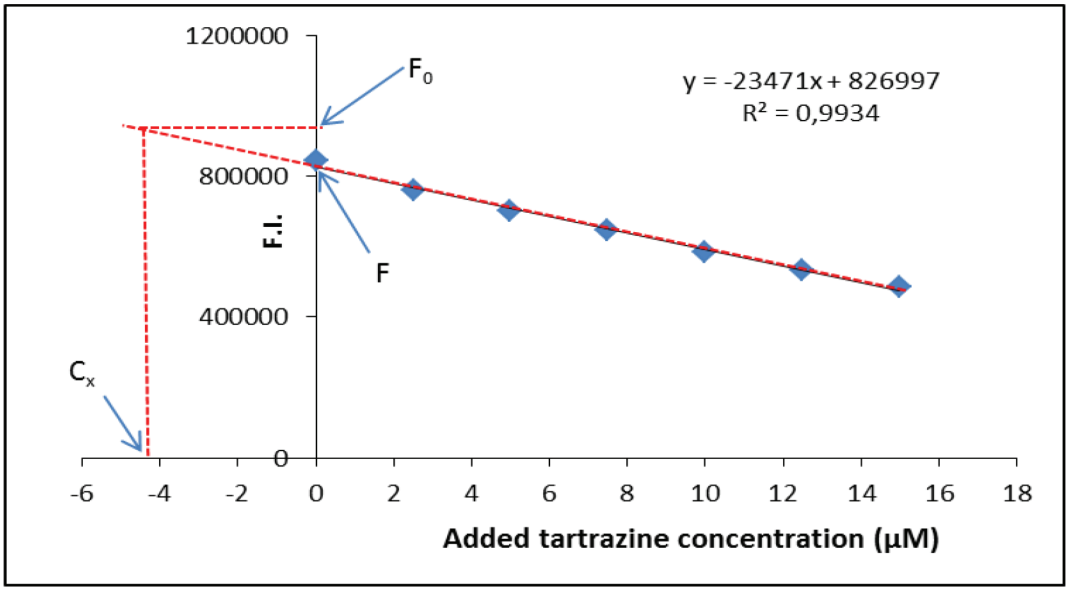

Figure S4. Standard addition graph for tartrazine determination (4.2 μM) in the cookie sample with CDs.

the static quenching process based on the complexation in the ground state should yield a linear Stern–Volmer plot.

Figure S5 shows the Stern–Volmer plot for the tartrazine-based quenching of the CDs. The I_0/I term linearly increased with the increasing tartrazine molar concentration until reaching 1.5×10^{-5} M. However, there was a positive deviation from the typically linear Stern–Volmer relationship after this concentration. This result showed that both static and dynamic quenching was observed in the system.

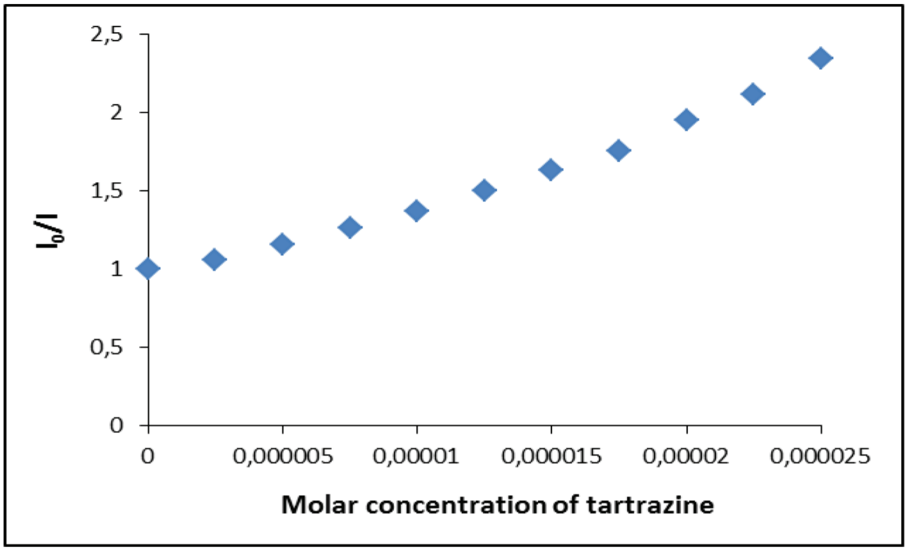

Figure S5. Stern–Volmer plot for the quenching of CDs by tartrazine.
